# Positive affect: phenotypic and etiologic associations with prosocial behaviors and internalizing problems in toddlers

**DOI:** 10.3389/fpsyg.2015.00416

**Published:** 2015-04-09

**Authors:** Manjie Wang, Kimberly J. Saudino

**Affiliations:** ^1^Psychology, Franklin and Marshall CollegeLancaster, PA, USA; ^2^Developmental Behavior Genetics Lab, Psychology, Boston UniversityBoston, MA, USA

**Keywords:** positive affect, prosocial behaviors, internalizing problems, genetics, environments

## Abstract

Despite evidence for the associations of positive affect to prosocial behaviors and internalizing problems, relatively little is known about the underlying etiology. The sample comprised over 300 twin pairs at age 3. Positive affect, prosocial behaviors, and internalizing problems were assessed using the Toddler Behavior Assessment Questionnaire (Goldsmith, 1996), the Revised Rutter Parent Scale for Preschool Children (Hogg et al., 1997), and the Child Behavior Checklist for ages 1.5–5 (Achenbach, 1991), respectively. Positive affect correlated positively with prosocial behaviors, and negatively with internalizing problems. Prosocial behaviors were negatively associated with internalizing problems. The relations of positive affect to prosocial behaviors and internalizing problems were due to environmental effects (shared and non-shared). In contrast, the link between prosocial behaviors and internalizing problems was entirely explained by genetic effects. The current study has moved beyond prior emphasis on negative affect and elucidated the less understood etiology underlying the associations between positive affect, prosocial behaviors, and internalizing problems. This study could guide the development of programs for promoting prosocial behaviors and alleviating internalizing problems in children.

## Introduction

### Positive affect: phenotypic and etiologic associations with prosocial behaviors and internalizing problems in toddler twins

Temperamental traits may predispose individuals to develop social competency, such as prosocial behaviors (Yagmurlu and Sanson, 2009); or psychopathology, such as internalizing problems (Nigg, 2006). Much of this research has focused on negative emotionality. In contrast, positive affect has been less explored in literature, although research has confirmed the independence of positive affect from negative affect (Putnam and Stifter, 2005). From a perspective of Positive Psychology, positive affect is of importance to promote favorable developmental outcomes, and prevent and alleviate psychological problems (Carr, 2011). Research has shown that positive affect is associated with prosocial behaviors (e.g., Lennon and Eisenberg, 1987) and fewer internalizing problems (e.g., Conway and McDonough, 2006; Zhou et al., 2009); however, there is a paucity of work that examines the genetic/environmental influences underlying these associations.

#### Positive affect: definition and etiology

Temperamental positive affect (PA) refers to a biologically based proneness to experience positive emotions (Watson and Naragon, 2009)^1^. Children with higher levels of PA are more inclined to experience and express cheerful emotions. PA is not simply equivalent to low negative affect (NA). PA has a connection with extraversion, whereas NA is linked with neuroticism in adults (Carr, 2011). Moreover, PA is different from NA evolutionarily and neurologically. From an evolutionary perspective, PA and NA are thought to be associated with different behavioral systems to carry out distinct evolutionary tasks (Watson and Naragon, 2009). Specifically, PA is strongly connected with the approach-oriented Behavioral Facilitation System that orientates individuals to experience pleasure and reward. In contrast, passive NA is closely tied to the withdrawal-oriented Behavioral Inhibition System that helps people to avoid undesirable outcomes. Research generally supports this, although some studies indicate that non-passive NA, such as anger and frustration, are related to the approach system (Rydell et al., 2003). Neurologically, research has suggested differential lateralization for processing positive and negative emotions (Davidson, 2003). Young infants display more brain activation in the left prefrontal cortex (PFC) when processing positive emotions, whereas the right PFC is more active when they process negative emotions (Fox and Davidson, 1988). This hemispheric specialization also has been suggested in adults (Davidson, 1984).

Both genetic and environmental factors influence individual differences in PA of both children (Goldsmith et al., 1999) and adults (Jang et al., 1998; Eid et al., 2003). Although shared environmental effects account for a moderate amount of the variation in PA in childhood (Goldsmith et al., 1997, 1999; Volbrecht et al., 2007), they have not been indicated in adulthood (Jang et al., 1998; Eid et al., 2003). This inconsistent finding may be attributed to age differences. Twins in childhood spend most of their time with their family, and shared family experiences contribute to the twin resemblances in PA; however, as they become adults and spend less time within the same environment, the environmental effects common to twins play a smaller role on PA.

#### Positive affect and prosocial behaviors

Prosocial behaviors refer to voluntary behaviors that can benefit others (Eisenberg and Fabes, 1998), and include helping, sharing, comforting, cooperating, donating, being fair and volunteering (Zahn-Waxler and Smith, 1992; Dunfield et al., 2011). These behaviors are motivated by empathy, an emotional reaction that enables people to understand and share the feelings of others (Spinrad and Eisenberg, 2009) and play an important role in the development of social and academic competence (Eisenberg et al., 1996; Spinrad and Eisenberg, 2009). For example, kindergarteners with higher levels of prosocial behaviors tend to have more mutual friendships and higher levels of peer acceptance, and subsequently have better classroom participation and school achievement (Ladd et al., 1999). The explanations for individual differences in prosocial behaviors could be social, evolutionary or biological. Social and situational factors that can influence prosocial behaviors include the interpretation of others' needs, the relationship to others, the reciprocal altruism, the number of bystanders, the normative pressure to help, and the evaluation of the cost to help (Batson, 1998). Evolutionally, biological relatedness and group selection are also possible factors explaining the prosocial behaviors (Eisenberg and Fabes, 1998). People are more likely to help genetically related individuals, which increases the likelihood of passing the same genes carried by the individuals. In addition, prosocial behaviors among group members may promote the survival of the group. Biological factors also play a role in prosocial behaviors. Brain areas involved in emotional systems such as amygdala and frontal cortex and the level of opioids in brain are related to prosocial behaviors (Eisenberg et al., 2006). Additionally, as is the case with PA, behavioral genetic studies have shown that individual differences in prosocial behaviors are attributed to genetic, shared environment, and non-shared environmental factors (Scourfield et al., 2004; Knafo and Plomin, 2006).

Prosocial behaviors have been linked to PA. Higher levels of PA lead to more sharing behaviors in children (Lennon and Eisenberg, 1987). Prosocial attitudes and behaviors in older adults predict their PA 3 years later after controlling for demographic characteristics (Kahana et al., 2013). The association between prosocial behaviors and PA might be mediated by the sense of self-efficacy. Prosocial behaviors could enable people to have a more positive view of themselves and enhance their self-efficacy, thus fostering PA (Fazio, 2009). PA, in turn, may increase people's sense of self-efficacy by yielding an optimistic view of their own abilities and resources to help people and then act out in a more prosocial manner (Cialdini et al., 1982). Although previous studies have focused on the link between prosocial behaviors and PA, relatively little is known about the genetic and environmental factors that underlie the association. An exception is the finding that the association between one aspect of prosocial behaviors, helping, and PA in early childhood is explained by both shared and non-shared environmental factors (Volbrecht et al., 2007). However, prosocial behaviors encompass a constellation of behaviors beyond helping, thus highlighting the need for research using a more comprehensive measure of prosociability which can provide a fuller understanding of the association between the two domains.

#### Positive affect and internalizing problems

There is also evidence of a negative relation between PA and internalizing problems in adolescence and adulthood, but findings for childhood are inconsistent. Lower levels of PA are associated with depression in adolescents (Phillips et al., 2002) and with more anxiety and depressive symptoms in adults (Kashdan, 2004; Naragon-Gainey et al., 2009). The relation of PA to internalizing problems is more complex in childhood. On one hand, higher PA (i.e., lower latencies to express positive emotions following challenge) is linked with fewer internalizing problems in early childhood (Conway and McDonough, 2006). Additionally, cross-cultural research has demonstrated that, in middle to late childhood, children with lower levels of PA (i.e., displaying positive emotions less frequently) have more internalizing problems in both China and the US (Zhou et al., 2009). On the other hand, children's intensity of positive emotions is not significantly related to internalizing problems at age 2 years (Putnam and Stifter, 2005). Accordingly, it seems that different indices of PA (latency, frequency, and intensity) may lead to different patterns of the relation between PA and internalizing problems.

Although previous studies have largely suggested a negative association between PA and internalizing problems, once again, relatively little is known about the underlying mechanisms of the association. The difficulties in the regulation of positive emotions may play a role in the psychopathology of mood and anxiety disorders (Weiss et al., 2015). Individuals with mood or anxiety disorders display maladaptive or inefficient regulation of positive emotions, specifically, giving inappropriate interpretation to and avoiding positive emotional states (Gilbert, 2012). The link between PA and internalizing problems might be mediated by executive functioning. Preschoolers with low levels of positive emotionality have been found to have problems with shifting attention (i.e., a domain of executive functioning), and those with shifting problems may be stubborn and inflexible in life and thus less likely to engage with environment and more likely to develop withdrawn problems (Ghassabian et al., 2014). Individual differences in internalizing problems have been attributed to genetic, shared, and non-shared environmental effects (Nikolas et al., 2013), which raises the question as to the extent to which its association with PA is genetically and/or environmentally mediated.

#### Prosocial behaviors and internalizing problems

Given that high PA is related to more prosocial behaviors and fewer internalizing problems, it is reasonable to expect a negative association between prosocial behaviors and internalizing problems and that this association may be mediated by PA. While prosocial behaviors have, in general, been linked to better mental health outcomes, the association between prosocial behaviors and internalizing problems is complex. Some research has found no significant association between prosocial behaviors and internalizing problems in children (Hay and Pawlby, 2003), whereas others looking at subgroups of children find that higher levels of prosocial behaviors is associated with both higher or lower levels of anxiety and depression from toddlerhood to late childhood (Nantel-Vivier et al., 2014). It has been proposed that the inconsistency within and across studies might reflect an optimum level of prosocial behavior, that is neither too high nor too low, is predictive of mental health in these domains. Moreover, factors such as parenting, age, and gender have been found to moderate the association between prosocial behaviors and internalizing problems (e.g., Gjerde and Block, 1991; Cáceda et al., 2014; Zarra-Nezhad et al., 2014). It has also been proposed that child temperament may play a role but this has not been examined (Nantel-Vivier et al., 2014).

Although investigation of potential moderators of the link between prosocial behaviors and internalizing problems are beyond the scope of the current study, our use of a genetically informative design elucidates the genetic and environmental mechanisms underlying the association. As mentioned earlier, both prosocial behaviors and internalizing problems are influenced by genetic and environmental factors, hence we hypothesized that the association between prosocial behaviors and internalizing problems would also be mediated by mutual genetic and/or environmental factors.

The current study moves beyond the prior emphasis on negative affect to elucidate the significance of PA on prosocial behaviors and internalizing problems in early childhood by examining the genetic/environmental etiology underlying these relations. By the end of toddlerhood, children are starting to experience more social interactions with parents and peers, display PA socially with increasing awareness of the meaning of PA of themselves and others (Messinger, 2008). At the same time, children in the transition from infancy to preschool begin to display empathetic concern and prosocial behavior with the development of understanding other people's perspectives (Eisenberg et al., 2006). Even at this young age, children with certain characteristics in temperament, such as lack of PA, may be at higher risk for internalizing problems (Campbell, 2006). Understanding how PA is tied to other facets of child behavior can enrich current knowledge of how emotionality more generally (i.e., not only negative affect) contributes to developmental outcomes. Such an understanding may serve to inform strategies of prevention and intervention.

## Method

### Sample

The Boston University Twin Project sample was recruited from birth records supplied by the Massachusetts Registry of Vital Records. All procedures were approved by the Boston University Institutional Review Board. As is standard for twin research, twins were selected preferentially for higher birth weight and gestational age. No twins with birth weights less than 1750 g or with gestational ages less than 34 weeks were included in the study. The sample included 304 same-sex twin pairs (140 Monozygotic and 164 Dizygotic; mean age = 2.99 years, *SD* = 0.08). Ethnicity was generally representative of the Massachusetts population (85.4% Caucasian, 3.2% Black, 2% Asian, 7.3% Mixed, 2.2% Other). Socioeconomic status according to the Hollingshead Four Factor Index (1975) ranged from low to upper middle class (range = 20.5–66; *M* = 50.9, *SD* = 14.1). Zygosity was determined via DNA analyses using DNA obtained from cheek swab samples. In the cases where DNA was not available (*n* = 3), zygosity was determined using parents' responses on physical similarity questionnaires which have been shown to be more than 95% accurate when compared to DNA markers (Price et al., 2000). After obtaining informed consent, parents (94% mothers) completed questionnaires (see below) regarding child temperament and behavior problems.

### Measures

#### Positive affect (PA)

PA was assessed on the Pleasure subscale from the Toddler Behavior Assessment Questionnaire (Goldsmith, 1996). This subscale consists of 10 questions regarding the child's frequency of smiling, laughing, or squealing with joy in specific situations (e.g., playing with favorite toys). Parents (94% mothers) indicated on a 7-point scale how frequently the child demonstrated the behavior during the previous month (1 = “*Never*” to 7 = “*Always*”). In our sample, internal consistency as indicated by Cronbach's alpha was 0.75.

#### Prosocial behaviors

Prosocial behaviors were assessed using the prosocial subscale of the Revised Rutter Parent Scale for Preschool Children (Hogg et al., 1997). This subscale consists of 11 items and evaluates a variety of prosocial behaviors including fairness, empathy, volunteering, helping, kindness, comforting, cooperating, resolving conflicts, and sharing (e.g., “tries to be fair in games,” “helps other children who are feeling ill,” “shares out treats with friends”). Parents were asked to indicate on a 3-point scale (0 = “*not true*”; 1 = “*somewhat true*”; 2 = “*definitely true*”) how well each item described the child's behavior in the past 6 months. The prosocial subscale showed good internal consistency in our sample (α = 0.81).

#### Internalizing problems

Internalizing problems were assessed using the Child Behavior Checklist for ages 1.5–5 (CBCL/1.5–5; Achenbach, 1991). The internalizing problems subscale consists of 36 items assessing 4 behavioral syndromes (i.e., Anxious/Depressed, Emotionally Reactive, Somatic Complaints, and Withdrawn). Parents were asked to indicate on a 3-point scale how well each item described their children's behavior within the past 2 months (0 = “*not true of their child*,” 1 = “*somewhat or sometimes true*,” 2 = “*very true or often true*”). The possible range for internalizing problems was 0–72. In our sample scores ranged from 0 to 33. The internal consistency for the internalizing problems subscale in our sample was 0.84.

### Statistical analyses

#### Data transformations

Internalizing problem scores were positively skewed and were normalized using the BLOM transformation in the SAS RANK procedure. Because twin correlations can be inflated by variance due to gender, all three scores were residualized for gender effects (McGue and Bouchard, 1984). These residualized scores were used in all behavioral genetic analyses.

#### Correlational analyses

Twin intraclass correlations were calculated as indices of indicating co-twin similarity. When MZ twins are more similar than DZ twins, genetic influences are indicated. When the DZ intraclass correlation exceeds one-half the MZ correlation, shared environmental effects are suggested. To evaluate genetic and environmental contributions to the phenotypic correlation between variables, cross-twin cross-variable correlations were calculated. For example, for the association between positive affect and internalizing problems, the cross correlation involved correlating the score of Twin A for positive affect with score of Twin B for internalizing problems, and vice versa. Cross correlations are the essence of a multivariate analysis of covariance. Genetic contributions to the covariance between variables are indicated when the MZ cross correlation is greater than the DZ cross correlation. If the DZ cross correlation is greater than one-half the MZ cross correlation, shared environmental effects may influence the association between phenotypes.

#### Model-fitting analyses

**Figure 1:**
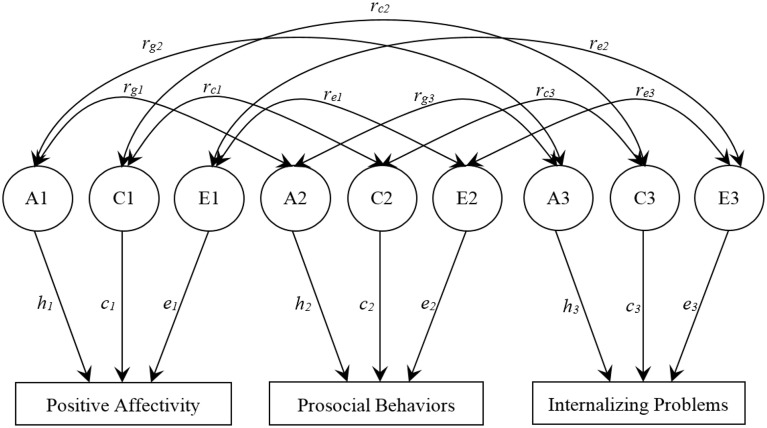
**Trivariate Correlated Factors Model**. The full model includes additive genetic (A), shared environmental (C), and non-shared environmental (E) factors. The path coefficients, *h, c*, and *e*, are standardized partial regression coefficients indicating the relative influence of the latent factors on the phenotypes. *r_g_, r_c_*, and *r_e_* represent the genetic, shared-environmental, and non-shared environmental correlations, respectively.

A trivariate correlated factors model was used to examine the extent to which genetic (A), shared environmental (C), and non-shared environmental (E) factors accounted for the variances of PA, prosocial behaviors, and internalizing problems, and the covariances between variables (see Figure 1). The latent factors A1, C1, and E1 refer to the genetic (additive), shared, and non-shared environmental influences on PA; A2, C2, and E2 to the genetic and environmental influences on prosocial behaviors; A3, C3, and E3 to the genetic and environmental influences on internalizing problems. The path coefficients, *h, c*, and *e*, are standardized partial regressions indicating the relative influence of the latent factors on the phenotypes. The square of these path coefficients estimates the genetic and environmental variances for each phenotype. Of particular interest in this model are the estimated parameters *r_g_, r_c_*, and *r_e_* (i.e., the genetic, shared environmental, and non-shared environmental correlations, respectively, between phenotypes). The genetic correlation indicates the extent to which genetic effects on one phenotype correlates with genetic effects on another, *independent of the heritability of each phenotype*. The genetic factors that influence two phenotypes can covary perfectly even though the genetic effects on each phenotype contribute only slightly to the phenotypic variance. Thus, *r_g_* can be 1.0 even though the genetic contribution to the phenotypic correlation is only modest if the heritability of each phenotype is modest and the same genetic effects operate on each phenotype. Conversely, two phenotypes may be substantially heritable, but the genetic correlation would be zero if the genetic effects on the two phenotypes do not overlap. Similar logic applies to *r_c_* and *r_e_*.

In addition to the full model, several reduced models with specific correlation paths fixed to zero were also tested. Models were fit to raw data using a maximum likelihood pedigree approach implemented in Mx structural equation modeling software (Neale et al., 2003). This approach allows the inclusion of participants with incomplete data. The overall fit of a model can be assessed by calculating twice the difference between the negative log-likelihood (-2LL) of the model and that of a saturated model (i.e., a model in which the variance/covariance structure is not estimated and all variances and covariances for MZ and DZ twins are estimated). The difference in -2LL is asymptotically distributed as χ^2^ with degrees of freedom equal to the difference in the number of parameters in the full model and that in the saturated model. In addition, a standard fit index, Akakie's information criterion (AIC; AIC = Δχ^2^ −2^*^Δ*df*) was used to assess models' fits (Neale and Cardon, 1992). Negative AIC values indicate good fit of the model to the observed data, and the model that minimizes AIC is a better-fitting model (Akaike, 1987). Because the reduced models were nested in the full model, the *relative* fit of the reduced model was determined by the χ^2^ difference (Δχ^2^) between full model and the reduced model, and corresponding change in degrees of freedom (Δ*df*). A significant Δχ^2^ indicates that the parameter not included in the reduced model could not be dropped without a significant decrement in fit and is therefore, significant.

## Results

### Descriptive statistics

Means and standard deviations of PA, prosocial behaviors, and internalizing problems by gender and zygosity are presented in Table 1. We evaluated mean differences for gender and twin type using generalized estimating equations (GEE) implemented in the SAS GENMOD procedure to account for dependence in the data due to the fact that our sample comprised pairs of twins. GEE are an extension of the standard generalized linear models that allow modeling of correlated data (Liang and Zeger, 1986; Zeger and Liang, 1986). For all three variables, the main effects of gender and zygosity, and the gender × zygosity interactions were non-significant.

**Table 1:** **Untransformed means (SD) at age 3 by sex and zygosity**.

**Age**	**Males**		**Females**		**Effect**					
	**MZ twins**	**DZ twins**	**MZ twins**	**DZ twins**	**Gender**		**Zygosity**		**Gender × Zygosity**	
					***z***	***p***	***z***	***p***	***z***	***p***
Positive affect	55.84 (6.76)	56.40 (7.35)	56.41 (6.40)	55.70 (7.33)	0.79	0.43	0.79	0.43	−0.86	0.39
*n*	133	175	133	141						
Prosocial behaviors	14.69 (3.57)	15.17 (3.74)	15.64 (3.53)	15.82 (3.77)	1.02	0.31	0.66	0.51	−0.39	0.69
*n*	136	175	133	146						
Internalizing problems	5.92 (4.99)	7.00 (6.11)	5.18 (4.34)	7.44 (5.44)	−1.13	0.26	−0.05	0.96	1.08	0.28
*n*	139	174	134	145						

### Correlations

PA correlated positively with prosocial behaviors (*r* = 0.29, *p* < 0.001) and negatively with internalizing problems (*r* = −0.28, *p* < 0.001). Prosocial behaviors and internalizing problems were negatively related (*r* = −0.14, *p* < 0.05). Twin intraclass correlations and cross-twin cross-variable correlations are presented in Table 2. For all three variables, the intraclass correlations for MZ twins exceeded those for DZ twins, suggesting genetic influences. In addition, the DZ correlations exceeded one-half the MZ correlation, suggesting that shared environmental effects also influence the three domains. For the relations of PA to the other two phenotypes, the MZ and DZ cross correlations were similar in magnitude and hint that shared environmental effects may contribute to the associations between PA and the other two phenotypes. For the association between prosocial behaviors and internalizing problems, the cross correlation for MZ twins was higher than that for DZ twins, suggesting that genetic effects may influence the phenotypic association, which can be tested by more powerful multivariate genetic model-fitting analyses.

**Table 2:** **Twin intraclass correlations and cross-twin cross-variable correlations**.

**Correlations**	**MZ twins**	**DZ twins**
**INTRACLASS CORRELATIONS**		
Positive affect	0.69^**^	0.62^**^
Prosocial behaviors	0.65^**^	0.44^**^
Internalizing problems	0.73^**^	0.53^**^
**CROSS-TWIN CROSS-VARIABLE CORRELATIONS**		
Positive affect–Prosocial behaviors	0.22^*^	0.22^*^
Positive affect–Internalizing problems	−0.18^*^	−0.22^*^
Prosocial behaviors–Internalizing problems	−0.13^*^	−0.04

** *p* < 0.01;

* *p < 0.05*.

### Model-fitting analyses

Table 3 presents the fit statistics for the model-fitting analyses. To determine sources of the covariance, models were fit where each type of the sources (i.e., genetic, shared or non-shared environmental correlation) was individually constrained to zero. A significant change in χ^2^ indicated that the parameter contributed significantly to the covariance and could not be dropped from the model. For the association between PA and prosocial behaviors, dropping the genetic correlation (*r_g1_*) did not cause a significant change in χ^2^ (*p* = 0.909); however, neither the shared (*r_c1_*) nor the non-shared environmental (*r_e1_*) correlation could be set to zero (*p* = 0.011, 0.012, respectively). Thus, environmental factors contribute to the association between PA and prosocial behaviors. For the association between PA and internalizing problems, it was possible to drop any one of the three sources of the covariance without a significant change in fit, but it was not possible to drop all three sources simultaneously (*p* = 0.000). Given that the model with no genetic correlation (*r_g2_*) yielded the smallest change in χ^2^ and the lowest AIC (−31.356), this model seems more plausible. After *r_g2_* was dropped, shared, and non-shared correlations become significant. For the association between prosocial behaviors and internalizing problems, dropping the genetic correlation (*r_g1_*) lead to a significant decrement in fit (*p* = 0.013), whereas the shared (*r_c3_*) or non-shared environmental (*r_e3_*) correlations could be dropped without significant change in fit (*p* = 0.367, 0.975, respectively). Taken together, the most parsimonious model is the one without *r_g1_, r_g2_, r_c3_*, and *r_e3_*, which fit data best (AIC = −36.632, *p* = 0.636).

**Table 3:** **Fit statistics for models**.

	**Overall fit of model^a^**						**Relative fit of model^b^**					
	**-2LL**	***df***	***χ ^2^***	**Δ*df_1_***	***p***	**AIC**	**Δ*χ*^2^**	**Δ*df_2_***	***p***
Saturated model	8232.326	1710							
Full model	8249.143	1734	16.817	24	0.856	−31.183			
Drop *r_g1_*	8249.156	1735	16.830	25	0.888	−33.170	0.013	1	0.909
Drop *r_c1_*	8255.568	1735	23.242	25	0.563	−26.758	6.425	1	0.011
Drop *r_e1_*	8255.504	1735	23.178	25	0.567	−26.822	6.361	1	0.012
Drop *r_g2_*	8250.970	1735	18.644	25	0.814	−31.356	1.827	1	0.176
Drop *r_c2_*	8251.802	1735	19.476	25	0.774	−30.524	2.666	1	0.103
Drop *r_e2_*	8252.546	1735	20.220	25	0.735	−29.780	3.403	1	0.065
Drop *r_g2_*+*r_c2_* + *r_e2_*	8292.624	1737	60.298	27	0.000	6.298	43.481	3	0.000
Drop *r_g3_*	8255.247	1735	22.921	25	0.582	−27.079	6.104	1	0.013
Drop *r_c3_*	8249.956	1735	17.630	25	0.858	−32.370	0.814	1	0.367
Drop *r_e3_*	8249.144	1735	16.818	25	0.888	−33.182	0.001	1	0.975
**Drop *r_g1_*+*r_g2_*+*r_c3_*+*r_e3_***	**8251.694**	**1738**	**19.368**	**28**	**0.886**	**−36.632**	**2.551**	**4**	**0.636**

*-2LL, log-likelihood statistic; df, degree of freedom; χ ^2^, chi-square fit statistic = -2LL difference between a model and the saturated model; Δdf_1_, degree of freedom difference between a model and the saturated model; AIC, Akaike's information criterion; Δχ^2^, chi-square difference between a reduced model and the full model; Δdf_2_, degree of freedom difference between a reduced model and the full model. The best-fitting model is indicated in bold*.

a *Overall fit of the model is determined by the difference in -2LL of each model and that of the saturated model*.

b *Relative fit of the model is determined by the χ ^2^ difference (Δχ^2^) between the full model and each reduced model*.

Estimates of genetic and environmental variances and correlations from the best-fitting models are presented in Table 4. Almost half of the variance (49%) in PA was influenced by shared environmental factors and the remaining variance was due to modest genetic (23%) and non-shared environmental (28%) factors. For both prosocial behaviors and internalizing problems, genetic effects played greater role accounting for over 40% of the variances in each. Shared environmental effects explained 20% and 31%, and non-shared environmental effects explained 32% and 25% of the variances in prosocial behaviors and internalizing problems, respectively.

**Table 4:** **Estimates (95% CI) from the best-fitting model**.

**Variance**	***h*^2^**	***c*^2^**	***e*^2^**
Positive affect	0.23 (0.02, 0.45)	0.49 (0.29, 0.65)	0.28 (0.22, 0.37)
Prosocial behaviors	0.47 (0.21, 0.66)	0.20 (0.05, 0.42)	0.32 (0.25, 0.42)
Internalizing problems	0.44 (0.23, 0.68)	0.31 (0.09, 0.49)	0.25 (0.19, 0.33)
**Covariance**	***r_g_***	***r_c_***	***r_e_***
Positive affect—Prosocial behaviors	–	0.75 (0.42, 1.00)	0.21 (0.07, 0.34)
Positive affect—Internalizing problems	–	−0.58 (−1.00, −0.33)	−0.22 (−0.35, −0.08)
Prosocial behaviors—Internalizing problems	−0.36 (−0.64, −0.15)	–	–

*h^2^, genetic variance; c^2^, shared environmental variance; e^2^, non-shared environmental variance. r_g_, r_c_, and r_e_ denote the genetic, shared environmental, and non-shared environmental correlations, respectively*.

Links between PA and both prosocial behaviors and internalizing problems were due to the environmental factors. The shared environmental correlation of 0.75 between PA and prosocial behaviors suggests that the shared environmental factors influencing the two phenotypes overlap substantially. These common shared environmental influences between PA and prosocial behaviors explained 78.8% of the phenotypic correlation (i.e., the phenotypic correlation between the two is largely due to shared environmental effects). In contrast, the correlation between shared environmental effects for PA and internalizing problems was more moderate (*r_c2_* = −0.58) and negative. Thus, shared environments that increase PA decrease internalizing problems, and vice versa. Again, these overlapping shared environmental factors largely explained the phenotypic association between PA and internalizing problems (i.e., 79.8%). Although more modest, the non-shared environmental correlations between PA and prosocial behaviors, and between PA and internalizing problems showed a similar pattern (i.e., positive between PA and prosocial behaviors, *r_e1_* = 0.21; and negative between PA and internalizing, *r_e2_* = −0.22). These overlapping non-shared environmental effects explained the remaining phenotypic covariance between PA and the other two variables (i.e., 21.2% between PA and prosocial behaviors, and 20.2% between PA and internalizing problems). Genetic factors contributed to only the association between prosocial behaviors and internalizing problems. The genetic correlation of −0.36 suggested a moderate genetic overlap across the two constructs; however, these common genetic effects exclusively explained the phenotypic correlation between the two domains.

## Discussion

The current study finds clear evidence for the phenotypic associations between PA and prosocial behaviors, and between PA and internalizing problems, as well as for the environmental factors contributing to these associations. The shared and non-shared environmental covariations underlying the phenotypic covariations highlight the importance of the social contexts in which children are reared with their siblings as well as the social environments in which they encounter unique social experiences. Family-wide or unique experienced environments, both of which may foster children's PA, may effectively promote prosocial behaviors and eliminate internalizing problems. Therefore, interventions designed to improving prosocial behaviors and internalizing problems could utilize the environmental contexts supporting children's development of PA.

### Positive affect and prosocial behaviors

Prior research has found that shared and non-shared environmental effects mediated the association between PA and helping (Volbrecht et al., 2007). The present study extends these findings to prosocial behavior more broadly defined (i.e., beyond helping), and thus supports the impact of environmental factors on general positive development of children. For young children, parenting behavior is a likely source of these environmental influences. Positive parenting, such as high maternal responsiveness, more parental induction and reasoning (i.e., parental practices providing explanations to the consequences of behaviors and fostering perspective taking in children), greater parental warmth and autonomy support, and parental modeling of prosocial behaviors could promote children's prosocial behaviors (Clark and Ladd, 2000; Eisenberg et al., 2006). Positive parenting also plays an important role in children's PA. Parents, who are more sensitive and responsive to infants' needs, and display more positive affect toward to infants, tend to have infants with higher levels of PA (Volling et al., 2002; Kochanska et al., 2004). Moreover, maternal personality may influence children's development of PA (Goldsmith et al., 1999). For example, mothers' openness is positively correlated with children's PA (Kochanska et al., 2004). Parents with higher levels of PA also have infants who express more positive emotions (Volling et al., 2002), but this could reflect genetic as well as shared environmental transmission. In addition to the impact on children's behaviors, parental personality influences positive parenting by moderating the effect of demographic risk. Parents with lower education and income levels and more children tend to have less positive parental behaviors, but this is only the case for parents who are less optimistic (Kochanska et al., 2007). Therefore, it is possible that parental personality could mediate the relation of positive parenting to children's positive behaviors.

### Positive affect and internalizing problems

Prior research exploring the link between PA and internalizing problems has produced mixed results. PA has been found to be negatively associated with internalizing problems in middle childhood (Zhou et al., 2009), but not in toddlers (Putnam and Stifter, 2005), suggesting possible age effects. However, there were different conceptualizations of PA (frequency vs. intensity) across these studies thereby confounding age with the operationalization of PA. Our finding that toddlers who displayed PA more frequently tended to have less internalizing problems is consistent with research in middle childhood and adds clarity to the literature by suggesting that it is the frequency, not intensity, of PA is related to internalizing behaviors. Thus, different conceptualizations of PA may be differentially informative about other aspects of child behavior and are not necessarily interchangeable.

The current study also provides further evidence for the environmental etiology of the association between PA and internalizing problems. Again, parenting behaviors probably serve as an environmental source of the covariation between PA and internalizing problems. Highly-disciplined or overprotective parenting styles both could precede the development of internalizing problems (Duchesne et al., 2010; Kiel and Buss, 2010). Parental practice also plays a role in PA as discussed above, and therefore, parenting behaviors could mediate the association between PA and internalizing problems.

Genetic factors did not significantly contribute to the link between PA and internalizing problems. Lateral activation asymmetries (i.e., the left-sided prefrontal activation for positive emotions vs. the right-sided prefrontal activation for negative emotions) may explain this finding. From a dimensional perspective, internalizing problems might be considered as the temperamental extreme of the negative affect, and children with the high levels of negative affect are prone to internalizing problems including anxiety and depression (Klein et al., 2012). Associated with different hemispheres, positive and negativity affect might be regulated by different genetic factors influencing the two hemispheres. Future work is needed to test this assumption.

### Prosocial behaviors and internalizing problems

Interestingly, the environmental factors influencing prosocial behaviors and internalizing problems were not correlated although they both overlapped with those on PA. In other words, some of the environmental effects on PA impact prosocial behaviors but not internalizing problems and vice versa. This could be the case given that different environmental influences have different effects on each aspect of children's socio-emotional behaviors. For instance, parental encouragement of children's emotion labeling influences children's development of prosocial behaviors above and beyond the effects of both children's age and parents' own emotion labeling and explanations on emotions during the interaction with children (Brownell et al., 2013). The amount of parental speech during the parent-child interaction when the child is 1-year-old predicts anxiety and mood disorders at age seven (Marwick et al., 2013). Accordingly, the quality or the content of parental speech could play a role in the development of prosocial behaviors, whereas the quantity of parental speech could influence the development of internalizing problems.

The genetic overlap between prosocial behaviors and internalizing problems is consistent with findings from molecular genetic research. The oxytocin receptor gene (*OXTR*) has been associated with both prosocial behaviors (Israel et al., 2009; Kogan et al., 2011) and internalizing problems (Costa et al., 2009). Oxytocin plays a role in both affiliation and emotion regulation (Donaldson and Young, 2008; Lee et al., 2009). Accordingly, it is possible that oxytocinergic system, where the *OXTR*-modulated oxytocin receptors express, is involved with both prosocial behaviors and internalizing problems. The genetic overlap between prosocial behaviors and internalizing problems has important implications for identifying other candidate genes. Future molecular genetics studies can target candidate genes for prosocial behaviors based on results of internalizing problems, and vice versa.

### Limitations and conclusion

The current findings should be evaluated in the context of some limitations. As is the case with most research looking at links between PA and prosocial behavior and internalizing problems in young children, the current study relied on parent ratings to assess all behaviors. This raises the possibility of a positivity bias whereby parents may be inclined to rate their children more favorably (e.g., high on both PA and prosocial behaviors, and low on internalizing problems). If this were the case we would expect to find shared environmental covariation between all phenotypes. However, the association between prosocial behaviors and internalizing problems arises solely due to genetic factors. This finding is important as prosocial behaviors would be more socially desirable and prone to positivity biases since they assess kindness and sharing which parents might view as more a reflection of their parenting than their children's tendencies for smiling and laughter. This differential pattern of genetic and environmental associations across variables suggests that the shared environmental covariance between PA and internalizing problems is not simply due to a positivity bias. Nonetheless, future research that employs multiple informants or methods is needed. Additionally, quantitative behavioral genetic analyses estimate the magnitudes of genetic and environmental variances of, and covariances between constructs, but do not identify the specific genetic or environmental factors. Nonetheless, the current findings highlight avenues for future research (i.e., the substantial shared environmental correlations between PA and both prosocial behavior and internalizing problems suggest looking at family-wide environments would be fruitful). Finally, the contemporaneous nature of the current study does not permit an analysis for the direction of the effects between phenotypes. It is possible that there may be a bidirectional relation between PA and the other constructs. Therefore, longitudinal studies are needed to explore the direction of the effects in future.

In conclusion, the current study is the first examination of the mechanisms underlying the associations among PA, prosocial behaviors (indicated by a diverse group of behaviors), and internalizing problems. Environmental influences contribute to the relations of PA to prosocial behaviors and internalizing problems. The association between prosocial behaviors and internalizing problems is explained by the genetic overlap. Our findings have both research and clinical implications.

### Conflict of interest statement

The authors declare that the research was conducted in the absence of any commercial or financial relationships that could be construed as a potential conflict of interest.

## Footnotes

^1^The definition of PA in the current paper emphasizes the specific facet of positive affect, or rather, the inclination to experience and express cheerful emotions, but does not reflect the higher-order level of well-being including social closeness, social potency, achievement-orientation, optimism, sociability, self-confidence conceptualized in the adult personality research.

## References

[B1a] Achenbach T. M. (1991). Manual for the Child Behavior Checklist 1 1/2–5. Burlington, VT: University of Vermont.

[B2a] Akaike H. (1987). Factor analysis and AIC. Psychometrika 52, 317–332 10.1007/BF02294359

[B1] Batson C. D. (1998). Altruism and prosocial behavior, in The Handbook of Social Psychology, 4th Edn., Vol. 2, eds Gilbert D. Fiske S. Lindzey G. (New York, NY: McGraw-Hill), 282–316.

[B2] Brownell C. Svetlova M. Anderson R. Nichols S. Drummond J. (2013). Socialization of early prosocial behavior: how parents talk about emotions is associated with sharing and helping in toddlers. Infancy 18, 91–119. 10.1111/j.1532-7078.2012.00125.x 23264753 PMC3524590

[B4] Cáceda R. Moskovciak T. Prendes-Alvarez S. Wojas J. Engel A. Wilker S. H.. (2014). Gender-specific effects of depression and suicidal ideation in prosocial behaviors. PLoS ONE 9:e108733. 10.1371/journal.pone.0108733 25259712 PMC4178187

[B5] Campbell S. B. (2006). Maladjustment in preschool children: a developmental psychopathology perspective, in Blackwell Handbook of Early Childhood Development, eds McCartney K. Phillips D. (Malden, MA: Blackwell Publishing), 358–378.

[B6] Carr A. (2011). Positive Psychology: The Science of Happiness and Human Strengths. Sussex: Routledge.

[B7] Cialdini R. B. Kenrick D. T. Baumann D. J. (1982). Effects of mood on prosocial behavior in children and adults, in The Development of Prosocial Behavior, ed Eisenberg N. (New York, NY: Academic Press), 339–359.

[B8] Clark K. E. Ladd G. W. (2000). Connectedness and autonomy support in parent-child relationships: links to children's socioemotional orientation and peer relationships. Dev. Psychol. 36, 485–498. 10.1037/0012-1649.36.4.485 10902700

[B9] Conway A. M. McDonough S. C. (2006). Emotional resilience in early childhood: developmental antecedents and relations to behavior problems. Ann. N.Y. Acad. Sci. 1094, 272–277. 10.1196/annals.1376.033 17347360

[B10] Costa B. Pini S. Gabelloni P. Abelli M. Lari L. Cardini A.. (2009). Oxytocin receptor polymorphisms and adult attachment style in patients with depression. Psychoneuroendocrinology 34, 1506–1514. 10.1016/j.psyneuen.2009.05.006 19515497

[B11] Davidson R. J. (1984). Affect, cognition and hemispheric specialization, in Emotion, Cognition and Behavior, eds Izard C. E. Kagan J. Zajonc R. (New York, NY: Cambridge University Press), 320–365.

[B12] Davidson R. J. (2003). Darwin and the neural bases of emotion and affective style. Ann. N.Y. Acad. Sci. 1000, 316–336. 10.1196/annals.1280.014 14766646

[B13] Donaldson Z. R. Young L. J. (2008). Oxytocin, vasopressin, and the neurogenetics of sociality. Science 322, 900–904. 10.1126/science.1158668 18988842

[B14] Duchesne S. Larose S. Vitaro F. Tremblay R. E. (2010). Trajectories of anxiety in a population sample of children: clarifying the role of children's behavioral characteristics and maternal parenting. Dev. Psychopathol. 22, 361–373. 10.1017/S0954579410000118 20423547

[B15] Dunfield K. Kuhlmeier V. A. O'Connell L. Kelley E. (2011). Examining the diversity of prosocial behavior: helping, sharing, and comforting in infancy. Infancy 16, 227–247 10.1111/j.1532-7078.2010.00041.x 32693496

[B16] Eid M. Riemann R. Angleitner A. Borkenau P. (2003). Sociability and positive emotionality: genetic and environmental contributions to the covariation between different facets of extraversion. J. Pers. 71, 319–346. 10.1111/1467-6494.7103003 12762418

[B17] Eisenberg N. Fabes R. A. (1998). Prosocial development, in Handbook of Child Psychology: Social, Emotional, and Personality Development, 5th Edn., Vol. 3, eds Damon W. (series ed.) Eisenberg N. (Vol. ed.) (New York, NY: Wiley), 701–778.

[B18] Eisenberg N. Fabes R. A. Karbon M. Murphy B. C. Wosinski M. Polazzi L.. (1996). The relations of children's dispositional prosocial behavior to emotionality, regulation, and social functioning. Child Dev. 67, 974–992. 10.2307/1131874 8706539

[B19] Eisenberg N. Fabes R. A. Spinrad T. (2006). Prosocial development, in Handbook of Child Psychology: Vol. 3. Social, Emotional, and Personality Development, 6th Edn, eds Eisenberg N.(Vol. ed.) Damon W. Lerner R. M. (series eds.) (Hoboken, NJ: Wiley), 646–718.

[B20] Fazio E. M. (2009). Sense of mattering in late life, in Advances in Conceptualization of the Stress Process, eds Ameshensel C. Schieman S. Wheaton B. (New York, NY: Springer), 149–176.

[B21] Fox N. A. Davidson R. J. (1988). Patterns of brain electrical activity during facial signs of emotion in 10-month-old infants. Dev. Psychol. 24, 230–236 10.1037/0012-1649.24.2.230

[B22] Ghassabian A. Székely E. Herba C. M. Jaddoe V. W. Hofman A. Oldehinkel A. J.. (2014). From positive emotionality to internalizing problems: the role of executive functioning in preschoolers. Eur. Child Adolesc. Psychiatry 23, 729–741. 10.1007/s00787-014-0542-y 24728546

[B23] Gilbert K. E. (2012). The neglected role of positive emotion in adolescent psychopathology. Clin. Psychol. Rev. 32, 467–481. 10.1016/j.cpr.2012.05.005 22710138

[B24] Gjerde P. F. Block J. (1991). Preadolescent antecedents of depressive symptomatology at age 18: a prospective study. J. Youth Adolesc. 20, 217–232. 10.1007/BF01537609 24265007

[B26a] Goldsmith H. H. (1996). Studying temperament via construction of the Toddler Behavior Assessment Questionnaire. Child Dev. 67, 218–235. 10.2307/1131697 8605830

[B25] Goldsmith H. H. Buss K. A. Lemery K. S. (1997). Toddler and childhood temperament: expanded content, stronger genetic evidence, new evidence for the importance of environment. Dev. Psychol. 33, 891–905. 10.1037/0012-1649.33.6.891 9383612

[B26] Goldsmith H. H. Lemery K. S. Buss K. A. Campos J. J. (1999). Genetic analyses of focal aspects of infant temperament. Dev. Psychol. 35, 972–985. 10.1037/0012-1649.35.4.972 10442866

[B27] Hay D. F. Pawlby S. (2003). Prosocial development in relation to children's and mothers' psychological problems. Child Dev. 74, 1314–1327. 10.1111/1467-8624.00609 14552400

[B28a] Hogg C. Rutter M. Richman N. (1997). Emotional and behavioural problems in children, in Child Psychology Portfolio, ed Insclare I. (Windsor, ON: NFER-Nelson), 1–13.

[B28] Israel S. Lerer E. Shalev I. Uzefovsky F. Riebold M. Laiba E.. (2009). The oxytocin receptor (OXTR) contributes to prosocial fund allocations in the dictator game and the social value orientations task. PLoS ONE 4:e5535. 10.1371/journal.pone.0005535 19461999 PMC2680041

[B29] Jang K. L. McCrae R. R. Angleitner A. Riemann R. Livesley W. J. (1998). Heritability of facet-level traits in a cross-cultural twin sample: support for a hierarchical model of personality. J. Pers. Soc. Psychol. 74, 1556–1565. 10.1037/0022-3514.74.6.1556 9654759

[B30] Kahana E. Bhatta T. Lovegreen L. D. Kahana B. Midlarsky E. (2013). Altruism, helping, and volunteering: pathways to well-being in late life. J. Aging Health 25, 159–187. 10.1177/0898264312469665 23324536 PMC3910233

[B31] Kashdan T. B. (2004). The neglected relationship between social interaction anxiety and hedonic deficits: differentiation from depressive symptoms. J. Anxiety Disord. 18, 719–730. 10.1016/j.janxdis.2003.08.001 15275949

[B32] Kiel E. J. Buss K. A. (2010). Maternal expectations for toddlers' reactions to novelty: relations of maternal internalizing symptoms and parenting dimensions to expectations and accuracy of expectations. Parent. Sci. Pract. 10, 202–218. 10.1080/15295190903290816 21037974 PMC2964838

[B33] Klein D. M. Dyson M. W. Kujawa A. J. Kotov R. (2012). Temperament and internalizing disorders, in Handbook of Temperament, eds Zentner M. Shiner R. L. (New York, NY: Guilford Press), 541–561.

[B34] Knafo A. Plomin R. (2006). Prosocial behavior from early to middle childhood: genetic and environmental influences on stability and change. Dev. Psychol. 42, 771–786. 10.1037/0012-1649.42.5.771 16953685

[B35] Kochanska G. Aksan N. Penney S. J. Boldt L. J. (2007). Parental personality as an inner resource that moderates the impact of ecological adversity on parenting. J. Pers. Soc. Psychol. 92, 136–150. 10.1037/0022-3514.92.1.13 17201548

[B36] Kochanska G. Friesenborg A. E. Lange L. A. Martel M. M. (2004). Parents' personality and infants' temperament as contributors to their emerging relationship. J. Pers. Soc. Psychol. 86, 744–759. 10.1037/0022-3514.86.5.744 15161398

[B37] Kogan A. Saslow L. R. Impett E. A. Oveis C. Keltner D. Rodrigues-Saturn S. (2011). Thin-slicing study of the oxytocin receptor (OXTR) gene and the evaluation and expression of the prosocial disposition. Proc. Natl. Acad. Sci. U.S.A. 108, 19189–19192. 10.1073/pnas.1112658108 22084107 PMC3228468

[B38] Ladd G. W. Birch S. H. Buhs E. S. (1999). Children's social and scholastic lives: related spheres of influence? Child Dev. 70, 1373–1400. 10621962 10.1111/1467-8624.00101

[B39] Lee H. J. Macbeth A. H. Pagani J. H. Young W. S. III. (2009). Oxytocin: the great facilitator of life. Prog. Neurobiol. 88, 127–151. 10.1016/j.pneurobio.2009.04.001 19482229 PMC2689929

[B40] Lennon R. Eisenberg N. (1987). Emotional displays associated with preschoolers' prosocial behavior. Child Dev. 58, 992–1000. 10.2307/1130540 3608668

[B40a] Liang K. Y. Zeger S. L. (1986). Longitudinal data analysis using generalized linear models. Biometrika 73, 13–22 10.2307/2336267

[B41] Marwick H. Doolin O. Allely C. S. McConnachie A. Johnson P. Puckering C.. (2013). Predictors of diagnosis of child psychiatric disorder in adult-infant social-communicative interaction at 12 months. Res. Dev. Disabil. 34, 562–572. 10.1016/j.ridd.2012.09.007 23123869

[B41a] McGue M. Bouchard T. J. Jr. (1984). Adjustment of twin data for the effects of age and sex. Behav. Genet. 14, 325–343. 10.1007/BF01080045 6542356

[B42] Messinger D. (2008). Smiling, in Encyclopedia of Infant and Early Childhood Development, Vol. 3, eds Haith M. M. Benson J. B. (Oxford: Elsevier), 186–198. Republished in Social and Emotional Development in Infancy and Early Childhood.

[B43] Nantel-Vivier A. Pihl R. O. Coté S. Tremblay R. E. (2014). Developmental association of prosocial behaviour with aggression, anxiety and depression from infancy to preadolescence. J. Child Psychol. Psychiatry 55, 1135–1144. 10.1111/jcpp.12235 24762335

[B44] Naragon-Gainey K. Watson D. Markon K. E. (2009). Differential relations of depression and social anxiety symptoms to the facets of extraversion/positive emotionality. J. Abnorm. Psychol. 118, 299–310. 10.1037/a0015637 19413405 PMC2794796

[B45a] Neale M. C. Boker S. M. Xie G. Maes H. H. (2003). Mx: Statistical Modeling, 6th Edn. Richmond, VA: Department of Psychiatry.

[B80] Neale M. C. Cardon L. R. (1992). Methodology for Genetic Studies of Twins and Families. Dordrecht: Kluwer Academic Publishers.

[B45] Nigg J. (2006). Temperament and developmental psychopathology. J. Child Psychol. Psychiatry 47, 395–422. 10.1111/j.1469-7610.2006.01612.x 16492265

[B46] Nikolas M. Klump K. L. Burt S. A. (2013). Etiological contributions to the covariation between children's perceptions of inter-parental conflict and child behavioral problems. J. Abnorm. Child Psychol. 41, 239–251. 10.1007/s10802-012-9679-7 22996155 PMC3543475

[B47] Phillips B. M. Lonigan C. J. Driscoll K. Hooe E. S. (2002). Positive and negative affectivity in children: a multitrait– multimethod investigation. J. Clin. Child Adolesc. Psychol. 31, 465–479. 10.1207/S15374424JCCP3104_6 12402566

[B47a] Price T. S. Freeman B. Craig I. Petrill S. A. Ebersole L. Plomin R. (2000). Infant zygosity can be assigned by parent questionnaire data. Twin Res. 3, 129–133. 10.1375/136905200320565391 11035484

[B48] Putnam S. P. Stifter C. A. (2005). Behavioral approach-inhibition in toddlers: prediction from infancy, positive and negative affective components, and relations with behavior problems. Child Dev. 76, 212–226. 10.1111/j.1467-8624.2005.00840.x 15693768

[B48a] Rydell A. M. Berlin L. Bohlin G. (2003). Emotionality, emotion regulation, and adaptation among 5- to 8-year-old children. Emotion 3, 30–47. 10.1037/1528-3542.3.1.30 12899315

[B49] Scourfield J. John B. Martin N. McGuffin P. (2004). The development of prosocial behaviour in children and adolescents: a twin study. J. Child Psychol. Psychiatry 45, 927–935. 10.1111/j.1469-7610.2004.t01-1-00286.x 15225336

[B50] Spinrad T. L. Eisenberg N. (2009). Empathy, prosocial behavior, and positive development in the schools, in Handbook of Positive Psychology in Schools, eds Gilman R. Huebner E. S. Furlong M. J. (New York, NY: Routledge/Taylor & Francis Group), 119–129.

[B51] Volbrecht M. M. Lemery-Chalfant K. Aksan N. Zahn-Waxler C. Goldsmith H. H. (2007). Examining the familial link between positive affect and empathy development in the second year. J. Genet. Psychol. 168, 105–129. 10.3200/GNTP.168.2.105-130 17936968 PMC3197271

[B52] Volling B. L. McElwain N. L. Notaro P. C. Herrera C. (2002). Parents' emotional availability and infant emotional competence: predictors of parent-infant attachment and emerging self-regulation. J. Family Psychol. 16, 447–465. 10.1037/0893-3200.16.4.447 12561291

[B53] Watson D. Naragon K. (2009). Positive affectivity: the disposition to experience pleasurable emotional states, in The Handbook of Positive Psychology, 2nd Edn., eds Snyder C. R. Lopez S. J. (New York, NY: Oxford University Press), 207–215.

[B54] Weiss N. H. Gratz K. L. Lavender J. M. (2015). Factor structure and initial validation of a multidimensional measure of difficulties in the regulation of positive emotions: the DERS-positive. Behav. Modif. [Epub ahead of print]. 10.1177/0145445514566504 25576185 PMC4420643

[B55] Yagmurlu B. Sanson A. (2009). Parenting and temperament as predictors of prosocial behaviour in Australian and Turkish Australian children. Aust. J. Psychol. 61, 77–88 10.1080/00049530802001338

[B56] Zahn-Waxler C. Smith K. D. (1992). The development of prosocial behavior, in Handbook of Social Development, eds Van Hasselt V. B. Hersen M. (New York, NY: Plenum Press), 229–256.

[B57] Zarra-Nezhad M. Kiuru N. Aunola K. Zarra-Nezhad M. Ahonen T. Poikkeus A. M.. (2014). Social withdrawal in children moderates the association between parenting styles and the children's own socioemotional development. J. Child Psychol. Psychiatry 55, 1260–1209. 10.1111/jcpp.12251 24827990

[B58a] Zeger S. L. Liang K. Y. (1986). Longitudinal data analysis for discrete and continuous outcomes. Biometrics 42, 121–130. 10.2307/2531248 3719049

[B58] Zhou Q. Lengua L. J. Wang Y. (2009). The relations of temperament reactivity and effortful control to children's adjustment problems in China and the United States. Dev. Psychol. 45, 724–739. 10.1037/a0013776 19413428 PMC4080919

